# Metachronous colorectal cancer risk for mismatch repair gene mutation carriers – the advantage of more extensive surgery

**DOI:** 10.1186/1897-4287-9-S1-O1

**Published:** 2011-03-10

**Authors:** Susan Parry, Aung K Win, Finlay A Macrae, Bryan Parry, Lyle C Gurrin, Noralane M Lindor, Steven Gallinger, John L Hopper, Mark A Jenkins

**Affiliations:** 1 New Zealand Familial GI Cancer Registry, Genetic Services, Auckland City Hospital, New Zealand; 2 Department of Gastroenterology, Middlemore Hospital, Auckland, New Zealand; 3 Centre for Molecular, Environmental, Genetic and Analytic Epidemiology, The University of Melbourne, Parkville, Victoria, Australia; 4 Colorectal Medicine and Genetics, Royal Melbourne Hospital, Parkville, Victoria, Australia; 5 Colorectal Surgical Unit, Auckland City Hospital, New Zealand; 6 Department of Medical Genetics, Mayo Clinic, Rochester, Minnesota, USA; 7 Mount Sinai Hospital, University of Toronto, Toronto, Ontario, Canada

## Background

Appropriate surgical management of colorectal cancers (CRC) in Lynch Syndrome patients, i.e. carriers of germline mutation in a mismatch repair (MMR) gene, is controversial. The decision to remove more or less of the colon requires the consideration of relatively high risk of metachronous CRC with the functional consequence of more extensive surgery. Our aim was to estimate and compare the risks of metachronous CRC for MMR gene mutation carriers following segmental versus extensive removal of colon for their first colon cancer.

## Materials and methods

Risk of metachronous CRC was estimated for 382 carriers of MMR gene (172 *MLH1*, 167 *MSH2*, 23 *MSH6* and 20 *PMS2*) mutations from the Colon Cancer Family Registry, who had colorectal surgery for their first colon cancer. Age-dependent cumulative risks were calculated using Kaplan-Meier method. Multivariate Cox proportional hazards regression was used to estimate the association between the length of bowel removed and metachronous CRC risk.

## Results

Of 50 individuals who had extensive (subtotal or total) colectomy for first colon cancer, none were diagnosed with metachronous CRC over 414 person-years (incidence rate, 95% confidence interval, CI 0–7.2 per 1000 person-years). Of 332 individuals who had segmental colorectal resection for first colon cancer, there were 79 (24%) diagnoses of metachronous CRC over 3,131 person-years (incidence rate 25.2 per 1000 person-years; 95%CI 20.2–31.5 per 1000 person-years). This incidence rate was statistically different from that for individuals who had extensive surgery (*P* <0.001). Cumulative risk of metachronous CRC was 20% (95%CI 15–26%) at 10 years, 44% (95%CI 35–55%) at 20 years and 66% (95%CI 52–78%) at 30 years after segmental colectomy for a first colon cancer. Risk of metachronous CRC reduced by 24% (95%CI 4–40%; *P* 0.02) for every 10 cm of bowel removed. There was no difference in the frequency of colonoscopy after surgery for first colon cancer between extensive and segmental resection (one colonoscopy per 16 (95%CI 13–20) months after extensive resection compared to one colonoscopy per 20 (95%CI 18–21) months after segmental resection; *P* 0.2).

## Conclusions

Lynch Syndrome patients with colon cancer who have had a segmental colorectal resection have a high cumulative risk for a metachronous CRC. Patients with more extensive resection of the first colon cancer had a lower risk of metachronous CRC compared to less extensive surgery. These findings will better inform decision making about the extent of primary surgical resection.

